# Where AIRE we now? Where AIRE we going?

**DOI:** 10.1097/ACI.0000000000001041

**Published:** 2024-10-23

**Authors:** Patrick Bez, Martina Ceraudo, Fabrizio Vianello, Marcello Rattazzi, Riccardo Scarpa

**Affiliations:** a Rare Diseases Referral Center, Internal Medicine 1, Ca’ Foncello Hospital, AULSS2 Marca Trevigiana, Treviso; b Deparment of Medicine (DIMED); c Hematology Unit, Department of Medicine (DIMED), University of Padua, Padua, Italy

**Keywords:** autoimmune regulator, autoimmune polyendocrinopathy-candidiasis-ectodermal dystrophy, autoimmune polyendocrine syndrome type 1, autoimmunity, immune tolerance

## Abstract

**Purpose of review:**

The purpose of the review is to describe the most recent advancement in understanding of the pivotal role of autoimmune regulator (*AIRE*) gene expression in central and peripheral tolerance, and the implications of its impairment in the genetic and pathogenesis of autoimmune polyendocrinopathy-candidiasis-ectodermal dystrophy (APECED) manifestations with insight into possible treatment options.

**Recent findings:**

AIRE gene expression has an important role of central and peripheral tolerance. Different AIRE gene mutations cause APECED, whereas polymorphisms and some variants may be implicated in development of other more frequently autoimmune diseases. Impaired negative T cell selection, reduction of T regulatory function, altered germinal center response, activated B cells and production of autoantibodies explain the development of autoimmunity in APECED. Recent data suggest that an excessive interferon-γ response may be the primer driver of the associated organ damage. Therefore, Janus kinase (JAK)-inhibitors may be promising therapies for treatment of broad spectrum of manifestations.

**Summary:**

AIRE has a pivotal role in immune tolerance. Disruption of this delicate equilibrium results in complex immune perturbation, ranging from severe autoimmunity, like APECED, to more common organ-specific disorders. Therefore, a deeper understanding of the correlation between AIRE function and clinical phenotype is warranted given the potential translational implication in clinical practice.

## INTRODUCTION

Autoimmune polyendocrinopathy-candidiasis-ectodermal dystrophy (APECED), known also as autoimmune polyendocrine syndrome type 1 (APS1), arises from damaging genetic germline mutations in the autoimmune regulator (*AIRE*) gene, leading to multiple autoimmune diseases in affected individuals. Since its discovery in 1997, studies on the AIRE gene have significantly advanced our understanding of fundamental aspects of human immunology. Specifically, these studies have been crucial in unraveling the basic mechanisms underlying the immune system's ability to distinguish between self and nonself. Indeed, AIRE plays a pivotal and distinctive role in the process of immunological tolerance by promoting the expression of a vast array of self-antigens within the thymus to the growing thymocytes [1].

The absence of AIRE expression in the thymus leads to a defect in the clonal deletion of autoreactive T cells, which ultimately results in severe multiorgan autoimmunity syndrome called APECED [2]. Given this characteristic feature, APECED is a condition classified as an inborn error of immunity, specifically within the group of diseases of the immune dysregulation [3].

We have progressively developed a deeper understanding of the complexity of this condition, encompassing the genetic landscape, immunological consequences, and phenotypic variability.

In this literature review we will account for the most recent developments. 

**Box 1:**
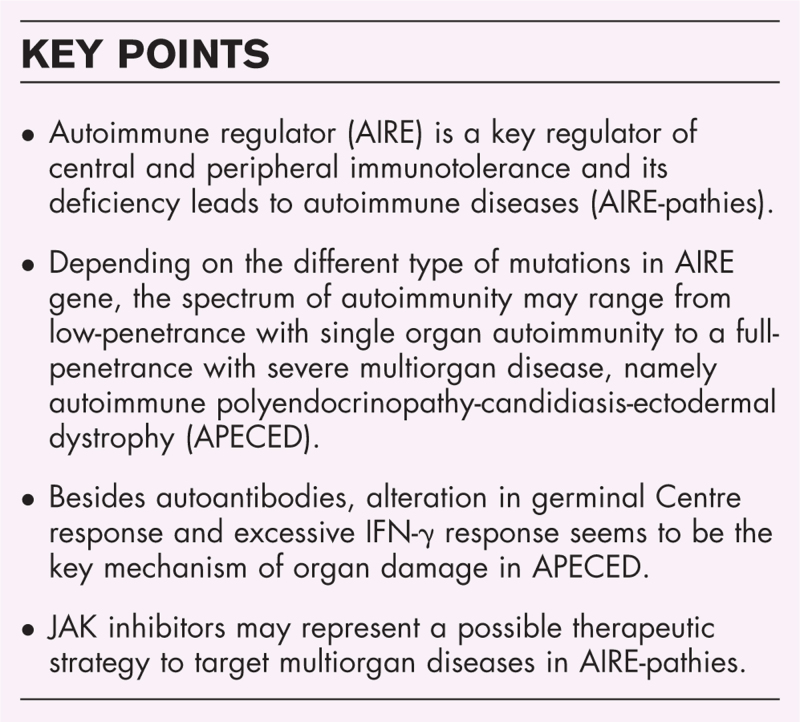
no caption available

## AIRE ROLE IN THYMUS AND PERIPHERY

**FIGURE 1:**
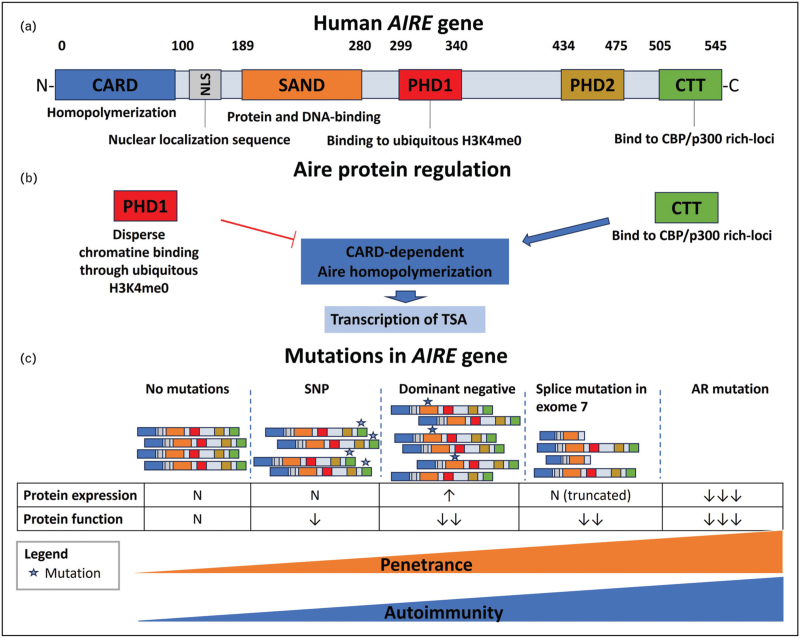
Human AIRE gene structure, regulation and mutations. (a) The structure and principal functions of protein domains of AIRE are depicted. (b) Aire functions depend on the tight regulation of CARD-dependent polymerization. In particular, CTT binds to CBP/p300 rich-loci and favours CARD-dependent polymerization of Aire, inducing transcription of tissue specific genes (TSA). On the other hand, PHD1 unspecific binding to chromatin (through H3K4me0) prevents Aire homopolymerization [8^▪▪^]. (c) Implications of different type of mutations on expression and function of the protein AIRE with consequent clinical implications. In absence of mutations, the immunological tolerance prevents autoimmunity. SNPs reduce function of AIRE which predispose to development of organ specific autoimmunity. In case of dominant negative mutations, augmented expression but reduced functionality is expected with development of a mild phenotype. In the case of exon 7 splice mutations, regular and truncated protein coexist with reduced function with a mild and late-onset phenotype. In case of autosomal recessive defects, the protein is frequently absent (or the function severely impaired) and patients develop a multiple organ autoimmunity. Abbreviations: CARD, caspase activation recruitment domain; SAND, named after Sp100, AIRE-1, NucP41/75, DEAF-1; PHD1, plant homeodomains 1; PHD2, plant homeodomains 2; CTT, C-terminal tail; CBP/p300, CREB-binding protein.

The human *AIRE* encoded protein is 545 amino acids long, has four LXXLL motifs and multiple major conserved domains: caspase activation recruitment domain (CARD), SAND (named after Sp100, AIRE-1, NucP41/75, DEAF-1) which is thought to be involved in DNA binding and protein-protein interaction, plant homeodomains 1 and 2 (PHD1 and PHD2), and C-terminal tail (CTT) [4] (Fig. 1a). In the animal model, CARD is involved in the homopolymerization of AIRE, and AIRE homopolymers manifest as nuclear condensates [5]. The process of homopolymerization is a necessary and tightly regulated event for *Aire* transcriptional activity. The nucleation signal is provided by transcriptional coactivators CBP/p300 clusters, which are prelocalized at enhancers target site and subsequently recruit AIRE, interacting with AIRE-CTT (Fig. 1b).

Once CARD has undergone polymerization, its polymer acts as a recruiting site for further Aire and CBP/p300 molecules, thereby creating a positive feedback loop for the assembly of transcriptional condensates, collecting multiple enhancers and reinforcing transcription. Conversely, the zinc-finger domain PHD1 specifically interacts with the widespread H3K4me0 marks in the genome [6,7], enabling AIRE to bind chromatin in a distributed unspecific manner and thereby counteract CBP/p300-driven CARD polymerization. The alternative chromatin interaction provided through PHD1 and CTT can therefore suppress or promote the formation of transcriptionally active condensates, influencing AIRE activity [8^▪▪^].

Once it binds to the chromatin, AIRE activates transcriptional elongation by a complex and not yet fully understood mechanism. Its interaction with bromodomain-containing protein 4 [9] likely allow the recruiting of positive transcription elongation factor b [10,11], along with DNA topoisomerases [12] and DNA damage repair proteins on the transcription site. The orchestrated activities of these enzymes induce modification of DNA topology with local chromatin relaxation [13] and the releasing of stalled RNA polymerase II, therefore enhancing mRNA processing to amplify tissue-specific antigens (TSA) gene expression. In this regard, a recent study alternatively suggested that the transient secondary structure Z-DNA may serve as anchor to recruit AIRE by enhancing double-stranded break generation and therefore facilitate promoter poising, rendering them more conducive to transcriptional upregulation; thus imply the transcriptional machineries are preassembled [14].

### Thymus

Within the thymus, AIRE is primarily localized to the nucleus of medullary thymic epithelial cells (mTECs) where it acts as an unusual transcriptional regulator able to enhance gene expression without relying on sequence-specific DNA promoter [14]. It interacts with chromatin at super-enhancer regions [15], stimulating local transcriptional activities and connecting disparate inter-chromosomal loci.

As a primary function, mTECs express a wide array of self-antigens in a promiscuous or ectopic manner, which are otherwise restricted to specific areas of the body [16]. It is now understood that AIRE partially controls the expression of many of these TSA in mTECs [17]. The mTEC individual expression of AIRE-inducible genes is highly variable with each mTEC expressing a mosaic of TSA [18]. Interestingly, the formation and stability of Z-DNA and the occurrence of double-stranded breaks, are stochastic events, which, along with the alternative PHD1 or CTT-mediated chromatin anchoring, might account for the varied expression of AIRE-inducible genes in individual mTECs. Therefore, AIRE is a crucial element in allowing thymocyte selection against TSA and in enforcing central T cell tolerance which ultimately results in the elimination of self- reactive T cells or their conversion to the Foxp3+ regulatory T cell (Treg) lineage.

Contrary to the earlier belief that AIRE solely functioned as a transcriptional regulator of TSA expression in mature mTECs, subsequent studies have revealed an even more complex role for AIRE in mTECs biology, particularly in mTECs maturation and differentiation themself. It has been indeed recognized that a subset of AIRE-expressing mTECs further differentiate into various post-AIRE subtypes that, while maintaining mTECs identity, closely resemble differentiated barrier epithelial cell types in peripheral tissues because of shared core transcriptional features. These cells were rapidly characterized and different gene signature were identified [19,20] like tuft cell, cornified keratinocyte, ciliated cell, neuroendocrine cell, ionocyte, myoepithelial, goblet-like, microfold-like, and enterocyte-like; collectively they were referred to as mimetic cells [21]. If these cells represent an additional way to increase antigen coverage within the thymic medulla to further shape lymphocyte development or constitute functional elements in tissue homeostasis is an area of ongoing investigation.

Even how AIRE contribute to mimetic cell developmental is still an unsolved question, but there is evidence of AIRE-dependent expression of transcription factors associated with embryonic plasticity of stem progenitor cells, including *Oct4*, *Sox2*, and *Nanog*[22]. It is thus raising the possibility that the effect of such transcription factors with so-called pioneering activity (i.e. transcription factors able to interact with nucleosome DNA, allowing them to access silent genes that other factors cannot reach) [23] may facilitate the mTECs fate changes trough cellular reprogramming towards mimetic cells. Overall, mTEC half-life is estimated to be 12–14 days and the post-AIRE half-life is estimated to be 7–8 days [24,25].

Whether the fascinating role of AIRE as a central regulator of immunological tolerance is progressively depicted, its specific locus regulation during the complex developmental dynamics of mTECs progenitor and during the life of the heterogeneous mTECs population is still uncertain.

Both noncanonical and canonical NF-κB signaling, particularly downstream of TNF superfamily receptors like RANK, are crucial for mTECs development and maintenance, including the AIRE-expressing compartment [26]. Single-positive CD4+ thymocytes are a key source of RANKL, a ligand essential for this process. NF-κB binds to the *Aire* promoter via a conserved noncoding sequence called CNS1 [27]. Various hematopoietic subsets, including thymic innate lymphoid cells, invariant T cell subsets, and B cells, contribute to the balance of TNF superfamily ligands, influencing mTECs development and lineage commitment. In addition, in a recent study Ikaros has been identified as a critical element for expansion and maintenance of AIRE-expressing mTECs as well as for their correct expression of TSA [28^▪^].

Beyond nuclear factor (NF)-κB and Ikaros signaling, additional transcriptional and posttranslational regulators are also involved is *Aire* expression modulation in mTECs. Epigenetic modifications, such as deacetylation by Sirtuin-1 [29], and direct transcriptional activation through the coordinated binding of Irf4, Irf8, Tbx21, and Tcf7 at the Aire transcriptional start site [30], have been demonstrated to influence *Aire* promoter function. Furthermore, JMJD6 has been identified as a critical factor in ensuring the correct splicing of Aire transcripts, which is essential for maintaining the appropriate abundance of the Aire protein [31]. In addition, posttranslational ubiquitination by FBXO3 regulates AIRE transcriptional activity [32] while HIPK2-mediated phosphorylation suppresses its coactivator activity [33].

### Periphery

*Aire* expression outside the thymus has been observed since its discovery [2,34]. Depending on the different methodological approaches used to detect its transcript or protein, initially *Aire* expression was most consistently demonstrated in peripheral lymphoid tissues (lymph nodes and spleen), and nonlymphoid tissues like testis [35]. In both humans and mice, AIRE protein in lymph nodes was mainly localized to myeloid migratory dendritic cells (DCs) subset [36,37]. DC are expected to contribute to tolerance induction in periphery and despite some evidence pointing at potential similarities between AIRE-mediated central and peripheral tolerance trough distinct TSA expression [38], some important early observation using bone marrow chimeras and thymus grafts challenge this hypothesis [17,39] and, recently, the dispensable role of Aire expression in CD11c+ DC for immune responses was confirmed [40]. Nevertheless, there is strong evidence that these cells can efficiently delete both neoantigen-specific CD4^+^ and CD8^+^ T cells in the periphery [38,41].

Further studies better specified at least three different cell types characterized by either *AIRE*-gene or -protein expression, identified using staining with anti CD11c and anti-EpCAM antibodies [42]. These rare hematopoietic populations in peripheral lymphoid tissues were collectively known as extrathymic AIRE-expressing cells (eTACs) and comprise of two distinct classes of antigen presenting cells (APCs): two subset of DC (CD11c^lo^ EpCAM^+^ and CD11c^lo^ EpCAM^−^) and a group 3 innate lymphoid cell (ILC3)-like cells (CD11c^-^ EpCAM^-^), that also express retinoic acid receptor-related orphan receptor γt (RORγt). While the DC subset was implicated in processes associated with peripheral tolerance, the AIRE+ ILC3-like cells were shown to participate in antifungal immune responses [43] by inducing Candida-specific T helper 17 (TH17) cell clones, therefore being immunogenic rather than tolerogenic cells. Importantly, since the production of TSA in AIRE+ ILC3 cells is not necessary for such antifungal responses [43], the data suggest that AIRE's role as a transcription regulator can be modulated depending on the cellular context. Moreover, recent studies that performed single cell RNAseq to trace cell fate, questioned a unique ontogeny as ILC3-like cells for the RORγt^+^AIRE^+^ eTACs [44], showing a blend of transcriptional programs shared with ILC3, mTECs, and myeloid cells, therefore suggesting a hybrid and a unique identity [45,46]. That feature resembles the expression of extrathymic lineage-defining transcriptional programs in mTECs mimetic cells [21]. It is of particular significance that the RORγt+AIRE+ eTACs lineage has additionally been proven to play a pivotal role in the generation of peripheral commensal-specific Tregs during the establishment of tolerance to commensal flora [45–47].

In summary, while a dualistic nature of thymic Aire-expressing mTECs is emerging, on one side rich sources of antigenic diversity themselves and on the other side precursors for a diverse range of differentiated cell types, the progressive understanding the transcriptional machinery and factor availability during mTECs differentiation is enhancing our knowledge on *Aire* locus regulation and on the broader transcriptional landscape affecting nascent AIRE-expressing cells. On the other hand, in the periphery, the growing body of data highlight the complexity of immune homeostasis, with extrathymic *Aire* expression involved in both antigen processing and presentation and generation of peripheral Treg, therefore with demonstrated effects on both proinflammatory and anti-inflammatory responses depending on context.

## GENETICS OF AUTOIMMUNE POLYENDOCRINOPATHY-CANDIDIASIS-ECTODERMAL DYSTROPHY

In light of the aforementioned complexity of AIRE's actions at the thymic and peripheral levels, it is unsurprising that mutations capable of modifying its protein's structure/function or reducing its availability at the nuclear level have significant and unpredictable phenotypic consequences.

The high variability in the clinical presentation observed among APECED patients across the countries [48,49,50^▪▪^], likely mirror the extended range of deleterious mutations that have been documented along the protein sequence, affecting all the functional domain.

APECED is classically autosomal recessive and, depending on the patient cohort, homozygous or compound heterozygous AIRE mutations are more often seen [48,49,50^▪▪^].

A clear genotype-phenotype correlation has not been established. Typically, patients develop a multitude of organ-specific autoimmune diseases over time, which often manifest during childhood. These include the well known endocrine disorders like hypoparathyroidism and autoimmune adrenal insufficiency, the “signature infectious manifestation” mucocutaneous candidiasis (MCC) and a range of other autoimmune features that can involve any organ [48,49].

In addition to biallelic recessive mutations, which are often fully ablative of AIRE protein expression, other genetic alterations have been identified that are capable of abolishing AIRE function to a variable degree and with incomplete phenotypic penetrance. This suggests a potential link between effective AIRE activity and associated autoimmunity (Fig. 1c).

Mutations occurring within splice site of intron 7, causing skipping of exon 7 and loss of the linking region between the SAND and PHD1 domains, determine atypical disease presentation with a late onset and slower progression in accumulating new disease components. Interestingly, this altered *AIRE* transcripts produced a protein with residual AIRE function with a normal nuclear localization, and that feature may contribute to prevent severe disease [51^▪^].

Dominant negative mutations in *AIRE* can also occur through missense mutations downstream of CARD that cluster within the PHD1, PHD2, and SAND domains and confer a variable hypomorphic effect on *AIRE* gene function [52]. Functionally, an augmented expression of dysfunctional AIRE protein with altered capacity to bind chromatin and induce gene expression has been found [53], typically resulting in an incompletely penetrant milder phenotype with later onset familial clustering, often masquerading as organ-specific autoimmunity [54].

Importantly, some of these *AIRE* mutations were found to a relatively high frequency in the general population and particularly in families with recurrent autoimmunity, suggesting that *AIRE* variants may modulate the phenotypic expression of common organ-specific autoimmune diseases.

Consistently, in GWAS (genome wide association studies), the *AIRE* p.Arg471Cys variant has been linked to an elevated risk of developing autoimmune Addison's disease [55], type 1 diabetes [56], and pernicious anemia [57]. Additionally, a coding variant in the SAND domain has been identified as a risk factor for rheumatoid arthritis [58].

It can thus be argued that several other AIRE-pathies (meaning that AIRE dysfunctions have a significant impact on) may exist in addition to APECED. Once again, the study of rare syndromes, such as APECED, is revealing important mechanisms that probably underlie the development of much more frequent diseases, and this may have far-reaching implications for larger populations in the future.

Nevertheless, some observations about the genetics of APECED patients suggest the potential involvement of additional genetic factors beyond *AIRE* in disease pathogenesis: clinical variability is evident [50^▪▪^] even within siblings with identical *AIRE* genetic variants [59], and in some cohorts, a significant proportion of patients lack biallelic *AIRE* mutations or deletions while exhibiting a clinical presentation indistinguishable from patients with biallelic *AIRE* mutations [49].

As previously stated, the control of the *AIRE* locus and protein expression has been the subject of investigation, resulting in the description of various regulators and interacting partners [29–33]. Mutations in some elements of this regulatory machinery may justify why some patients are clinically diagnosed with APECED despite a wild-type AIRE genotype.

## IMMUNOLOGICAL CONSEQUENCES OF AUTOIMMUNE REGULATOR DEFICIENCY

In the absence of AIRE, the thymic process of clonal deletion is defective, allowing auto-reactive T cells to escape in the periphery. Following loco regional lymph nodes germinal center reaction, T lymphocytes can match and help self-specific B cells, resulting autoimmune target organ infiltration, often with development of a wide range of autoantibodies with rare, if not unique, specificities. The resulting lymphocytic tissue infiltration cause progressive organ damage, composing the clinical picture of APECED [60].

In addition to AIRE's role in negative selection of self-reactive effector CD4^+^ T cells, AIRE has also been implicated in the diversion of distinct autoreactive T cell specificities into the Treg cell lineage [61]. In humans, the examination of lymph node specimens of APECED patients yielded evidence of a diminished overall frequency of Tregs and interactions between Tregs and nonfollicular T cells. Conversely, there was an expansion of the T follicular helper population (Tfh), which suggests an altered germinal center function [62^▪▪^]. Nonetheless, current evidence would suggest that the negative selection of effector T cells may play a more crucial role in pathogenesis over the positive selection of Tregs. Indeed, APECED patients do not have intrinsic defects in their Treg functionality [63^▪^], although they could be detected at lower frequency in peripheral blood and may present a skewed TCR repertoire [64].

The result of thymic selection is not limited to generate competent CD4^+^ effector T cells and Tregs. Among CD8^+^ T lymphocytes, APECED patients present an imbalanced TCR repertoire due to oligoclonal expansion of highly differentiated effector-type CD8^+^CD45RA^+^ T cell (probably corresponding to CD8^+^ effector memory re-expressing CD45RA, TEMRA) [65].

In an *Aire*-deficient mouse model, a significant accumulation of similarly activated and proliferating CD8^+^ and CD4^+^ T cell subsets was observed in the epithelial and submucosal tissue layers. Furthermore, a corresponding marked accumulation of CD4+ and CD8+ T cells was observed within the oral mucosa of APECED patients, even in the absence of oral candidiasis and RNA-sequencing data of oral mucosal tissue in five adult individuals with a history of MCC showed clear corroborative evidence of exaggerated type-1 responses. The pathogenic T cells secrete considerable quantities of interferon- γ (IFN-γ), which impairs the integrity of the mucosal barrier and results in the pathogenic growth of *C. albicans*[66].

In addition, recent evidence implicates γδ+ T cells in the development of ocular and pulmonary autoimmunity in the APECED mouse model. Interestingly, a corresponding expanded IL-17A+Vγ9+Vδ2+ T cell population was detected in the peripheral blood of APECED patients, suggesting the need for translational investigation [67].

Despite being primary recognized as a T cell driven disorder, APECED patients display a striking tendency to develop a broad array of autoantibodies against a multitude of tissue-specific antigen, enzymes and cytokines [68]. This is at least in part dependent from the T cell dysregulation, as the clearance of autoreactive B cell clones rely, in periphery, on intact Treg function [69–71]. In APECED patients, highly expanded autoreactive mature B cell clones with skewed repertoire are observed [68], including specific clonotypes to TSA expressed under AIRE's control in the thymus [72]. These findings, like the aforementioned increase in Tfh [62^▪▪^], suggest a disrupted regulation of the germinal center reaction, as the central B cell tolerance in APECED being considered unaltered [64]. Consistently, unbalanced B cell subpopulations towards terminally differentiated B cells [62^▪▪^] and an increase in soluble cytokines relevant for B cells homeostasis like BAFF [64] and APRIL [62^▪▪^] are reported in APECED patients. Conversely, B cells contribute to thymic function as relevant APC, contributing to T cell priming and expansion; furthermore, B cells can express AIRE themselves [73]. Therefore, the precise contribution of B cells in the autoimmune phenotype in APECED is still not fully elucidated.

From a clinical perspective, the detection of autoantibodies has proved invaluable in predicting the development of APECED specific disease components [74], although not with absolute certainty. The autoantibody repertoire is so extensive that virtually every one of the multiple manifestations of APECED must be considered autoimmune until proven otherwise. Furthermore, detection of high affinity autoantibodies from these patients has been a rich resource for the identification of novel autoantibody specificities that can explain the etiology of clinical features in diseases other than APECED, like the case of anti-Perilipin 1 in acquired generalized lipodystrophy [75]. Another example is the observation that the majority of APECED patients exhibit IgA autoantibodies against ameloblast-specific proteins, possibly explaining tooth enamel dysplasia, a prevalent and enigmatic feature of APECED [76].

The detection of autoantibodies directed against type I interferons represents an essential diagnostic tool for APECED, as they can be identified with high sensitivity during infancy, preceding the onset of clinical symptoms. Moreover, at this early age, these autoantibodies exhibit a high degree of specificity for APECED [77,78].

The identification of neutralizing autoantibodies against interleukin (IL)-17A, IL-17F and IL-22, which are critical cytokines involved in the mucosal defense against *Candida* spp. has been associated with the susceptibility to MCC in APECED patients, suggesting a potential autoimmune origin for this hallmark infectious manifestation [79,80]. In line with this hypothesis, the loss of extrathymic Aire in RORγt-lineage APC cells, which results in significant impairment of Candida-specific Th17 responses, may cooperate in the onset of MCC [43].

Even though it's an intriguing hypothesis, available data supporting a primary role of impaired type-17 immunity on Candida clearance are not fully convincing.

In addition, other recent data provided compelling evidence that pathogenic CD4^+^ and CD8^+^ T cells are major drivers fungal susceptibility at the mucosa, via impairing the integrity of the mucosal barrier through excessive production of interferon (IFN)-γ [66]. The further observation from mouse model that mucosal candidiasis was ameliorated by the inhibition of IFN-γ and/or Janus kinase (JAK)/STAT was recently translated into clinical, to evaluate the safety and efficacy of JAK/STAT inhibition in the management of in APECED patients, showing remarkably positive responses to ruxolitinib not only for MCC, but also for alopecia, nail dystrophy, keratitis, steroid-dependent autoimmune hepatitis, exocrine pancreatic insufficiency, renal potassium wasting, hypoparathyroidism, and diabetes insipidus [81^▪^]. This suggested that many other detrimental disease components could be mediated by excessive IFN-γ. The formal demonstration of this assumption has come recently, by showing that APECED patients had enhanced IFN-γ responses in blood and in multiple examined autoimmunity-affected tissues [82^▪▪^]. Similarly to a previous report, ruxolitinib treatment of five APECED patients led to clinical remission of multiple autoimmune manifestation like alopecia, oral candidiasis, nail dystrophy, gastritis, enteritis, arthritis, Sjögren's-like syndrome, urticaria, and thyroiditis and biochemical correction of the excessive IFN-γ signature [82^▪▪^]. Currently, a phase 2 trial of ruxolitinib in APECED for alopecia areata is recruiting patients (registration number: NCT05398809). These findings suggest that a clinical trial to assess the efficacy of JAK inhibitors in patients with APECED is warranted, as it could provide for the first time a broad-spectrum treatment for the various disease manifestations. It would be of interest to evaluate the long-term effects of treatment on the progression of the disease in cases where it is initiated at an early stage. In this respect, an early diagnosis, preceding the most severe manifestations, will be of great importance.

## CONCLUSION

Because of its unique function, *AIRE* has been the focus of extensive research efforts with the overall aim of improving our understanding of how autoimmunity can arise.

The results of this research have provided a more comprehensive insight into the underlying pathogenic mechanisms of APECED syndrome and effector branches.

Recently, another piece of the puzzle has been solved with the identification of one of the main mechanisms responsible for organ damage in APECED, namely the excessive IFN-γ response. Future clinical application will hopefully change our current medical management from a screening and treatment-based approach to a prevention-based intervention, thereby improving the quality of life of APECED patients.

## Acknowledgements

*None.*

### Financial support and sponsorship

*None.*

### Conflicts of interest

*There are no conflicts of interest.*

## REFERENCES AND RECOMMENDED READING

Papers of particular interest, published within the annual period of review, have been highlighted as:

▪ of special interest
▪▪ of outstanding interest
